# Determinants of COVID Vaccination Willingness among Health and Non-Health Studies Students: A Cross-Sectional Study

**DOI:** 10.3390/vaccines11050981

**Published:** 2023-05-15

**Authors:** Mario Marendić, Diana Aranza, Ivan Aranza, Dario Vrdoljak, Mario Podrug, Mirjana Milić

**Affiliations:** 1 University Department of Health Studies, University of Split, 21000 Split, Croatia; daranza@ozs.unist.hr (D.A.); mpodrug@ozs.unist.hr (M.P.); 2 University of Split School of Medicine, 21000 Split, Croatia; ivan.aranza@mefst.hr; 3 Faculty of Kinesiology, University of Split, 21000 Split, Croatia; darvrd@kifst.hr (D.V.); mirjanam@kifst.hr (M.M.)

**Keywords:** COVID-19, critical thinking, health information, health literacy, SARS-CoV-2, students, vaccination willingness

## Abstract

Students, as a relatively health-informed population group, may still have limitations in health literacy, which is a concern as students take increasing responsibility for their health and make independent health decisions. The aim of this study was to evaluate the overall attitudes towards COVID vaccination among university students and to investigate various factors contributing to vaccination willingness among health and non-health studies students. A total of 752 students from the University of Split were included in this cross-sectional study and completed a questionnaire that consisted of three sections: socio-demographic data, health status information, and information on vaccination against COVID-19. Results show that the majority of students of health and natural sciences were willing to be vaccinated, but the majority of students of social sciences were not (*p* < 0.001). Students who used credible sources of information had a more significant proportion of those willing to be vaccinated and the majority of students who used less credible sources (79%) or did not think about it (68.8%) were unwilling to be vaccinated (*p* < 0.001). Multiple binary logistic regression modeling shows that female gender, younger age, studying social sciences, negative opinion about the need to reintroduce lockdown and the effectiveness of epidemiological measures, and usage of less credible sources of information were the most important factors contributing to increased vaccination hesitancy. Therefore, improving health literacy and restoring trust in relevant institutions can be critical in health promotion and COVID-19 prevention.

## 1. Introduction

One of the most remarkable and significant disruptions in modern human history is the SARS-CoV-2 pandemic, which dramatically affected global development, caused a sharp increase in mortality, and impacted all aspects of society [1,2]. Since the outbreak of the COVID-19 pandemic, various countries around the world have taken significant steps to combat the disease [2]. In the spring of 2020, Croatia was one of the countries with the strictest epidemiological measures at the global level [3,4]. Although knowledge of the novel coronavirus has spread, epidemiological measures have occasionally loosened. However, despite the epidemiological efforts and the mandatory wearing of protective masks, the disease continues to spread worldwide [3]. Faced with the daily progression of the pandemic, authorities worldwide, in collaboration with scientists, scientific communities, and pharmaceutical companies, have invested efforts and economic research of the vaccine against COVID-19 [5].

The European Medicines Agency approved vaccines and ordered four types of vaccines (AstraZeneca/Oxford (adenoviral vector vaccine), Johnson & Johnson/Janssen (adenoviral vector vaccine), Pfizer/BioNTech (mRNA vaccine), and Moderna (mRNA vaccine)) [6], and the Croatian population vaccination officially began on 27 December 2020 [7]. Primarily, vaccinations were intended for people in poorer health, such as nursing home residents and people with disabilities. In addition, priority was given to health professionals, people over 65, and the chronically ill [6].

The abundance of health information that is readily available has a profound impact on both health-related decisions and behaviors. The importance of accuracy and trustworthiness in sources of health information has grown substantially in the pursuit of informed decisions that advance good health [8]. COVID-19 is a worldwide health crisis framed by countless misinformation and fake news that has its consequences and spreads even more distorted and misleading information [9,10]. Furthermore, scientists dealing with health information and health communication in public health think much of the communication during the COVID-19 pandemic was designed more to convince people of something instead of informing them [11]. However, anti-vaccine movements have increased recently and become even more vital during the pandemic [12,13]. Among the most common reasons for vaccine hesitancy are distrust towards government and pharmaceutical companies and concerns about side effects, all of which is additionally exacerbated by massive misinformation about the vaccines that could be found on social media [12,13,14,15]. Studies have shown that the most common reasons for vaccine hesitancy are insufficient confidence in the efficacy of vaccines and concerns about side effects [9,12,13].

Students, as a relatively health-informed population group, may still have limitations in health literacy, which is a concern as students take increasing responsibility for their health and make independent health decisions [16]. On the other hand, university students are considered a well-educated population that would explore their views on accepting new vaccinations because they are open-minded, educated, and should respond quickly to public health issues [17,18,19,20,21]. The World Health Organization has updated their definition of health literacy to emphasize the importance of using health information, not just understanding it. Furthermore, there is a clear emphasis on making informed decisions, both individually and as a community [22]. Health literacy played a significant role in saving lives during the global health crisis of COVID-19 [10,23].

Regardless of students’ future profession, it is essential today to have a good understanding of how to make responsible and safe decisions regarding health. Health students play a critical role in promoting health literacy and encouraging evidence-based medicine for informed decision making. The objective of this study was to evaluate the overall attitudes towards COVID vaccination among university students in Split, Croatia. Additionally, we sought to determine if future health professionals, who are enrolled in health-related studies, possess more knowledge about the effectiveness and significance of vaccines compared to non-health-related students. Furthermore, we aimed to determine if students from health-related studies are less susceptible to misinformation and more inclined to seek information from trustworthy sources. The study also examines other factors that contribute to vaccination willingness, in order to identify the set of most significant factors that reduce vaccination hesitancy.

## 2. Materials and Methods

### 2.1. Study Design and Setting

This cross-sectional study was conducted via an anonymous (online) survey using Google Forms in March 2021, during the 2020/2021 academic year. An invitation for participating in the study was sent to eight institutions which are a part of the University of Split, and four institutions responded affirmatively.

Associates from the faculty institutions contacted their students with a link to access an online survey. After the initial e-mail, reminders were sent three times at five-day intervals to reach the highest possible response rate.

The survey was anonymous, and before providing their responses, all students were informed about the purpose of the study and the purpose of the data collection. Therefore, there were no exclusion criteria.

#### 2.1.1. Participants

This study involved students from the University of Split’s four faculty institutions: the University Department of Health Studies, the Faculty of Science, the Faculty of Chemistry and Technology, and the Faculty of Humanities and Social Sciences.

For the purposes of data analysis, the faculty institutions were divided depending on the study area into Health Sciences (University Department of Health Studies), Natural Sciences (Faculty of Science, Faculty of Chemistry and Technology), and Social Sciences (Faculty of Humanities and Social Sciences).

#### 2.1.2. Questionnaire

For the purposes of this study, a new questionnaire was constructed. It consisted of 24 questions divided into three sections. The questionnaire was constructed based on a review of the available literature [8,24] and consultation with experts in the field of public health.

The first section included social–demographic factors such as age, gender, faculty institution, degree of study, place of residence (urban, semi-urban, village), marital status and living with others, as well as the method of attending classes (in person, online, combined, did not have classes).

The second section contained health status information: (i) presence of chronic disease such as diabetes or hypertension (possible responses were “yes” and “no”), (ii) recovery from COVID-19 infection (possible responses were “yes”, “no”, and “do not know”), (iii) description of symptoms in case participants had COVID-19 (possible responses were “asymptomatic disease”, “very mild symptoms”, such as a mild cold and no fever, “moderate symptoms”, such as increased body temperature (fever), “severe symptoms”, such as prolonged and severe fever, weakness and pain, “very severe symptoms”, such as requiring oxygen therapy, and “I did not have COVID-19”). For the purposes of analysis, symptoms of the disease were divided into four categories (no symptoms, milder form, more severe form, and not overcoming COVID-19).

The third section contained information on (i) vaccination against COVID-19 (possible responses were “yes”, “no”, and “I do not know”), (ii) trust in experts from the Civil Protection Directorate (a Likert scale was used, values ranged between 0 and 10, where 0 means “I do not trust at all”, and 10 means “I trust them entirely”), (iii) compliance with epidemiological measures (a Likert scale was used, values ranged between 0 and 10, where 0 means “I do not adhere at all”, and 10 means “I adhere to all epidemiological measures”), (iv) opinion about current epidemiological measures (possible responses were “yes”, “no”, and “I do not know”), (v) opinion on the need to reintroduce lockdown (possible responses were “yes”, “no”, and “I do not know”), and (vi) sources of information about COVID-19. For the purposes of analysis, the sources of information were divided into three groups: (i) credible sources of information (which included “Website of the Croatian Institute of Public Health (hzjz.hr)”, “Website of the Croatian Government (koronavirus.hr)”, “Scientific research articles”, “TV” (diary, information from the Civil Protection Directorate)), (ii) less credible sources of information (which included “The Internet (in general)”, “Newspapers”, “Articles on the Internet (webpages of Croatian news portals: 24 h, index.hr, dnevnik.hr, Slobodna Dalmacija, Jutarnji list, or some other internet sources)”, “Via friends”), and (iii) “I do not think about it”.

### 2.2. Data Analysis

Descriptive analysis was used to obtain frequency and percentage values for all variables. Likert-scale responses are presented as median with interquartile range (IQR). Differences between categorical variables were analyzed with the chi-square (χ^2^) test and Fisher exact test. Because of the statistically significant deviation of our data set from the Gaussian distribution, a nonparametric Kruskal–Wallis test by ranks was used to identify statistically significant differences between groups. In addition, multiple binary logistic regression was used to identify factors contributing to vaccination hesitancy. For the purposes of this analysis, participants were divided into two new subgroups. The first subgroup included all participants who were willing to be vaccinated, and the other subgroup included those who were not willing to be vaccinated and those who had not yet decided. The logistic regression model then predicted which factors significantly contributed to a participant being in the first subgroup, i.e., influenced the students’ decision to be vaccinated.

All responses received were coded and entered into an Excel spreadsheet. After creating the matrix, data were analyzed using IBM SPSS Statistics for Windows, Version 20.0. (Armonk, NY, USA: IBM Corp.), and the results were interpreted at a significance level of *p* < 0.05.

## 3. Results

This study involved 752, students with a response rate of 21.5% (N = 752/3490 of the students contacted). The sample size was calculated using the Raosoft online calculator [25]. With the student population from the University of Split size of 20,000 students, a confidence level of 95%, a margin of error of 5%, and a response distribution of 50%, this study’s minimum adequate sample size was 377. Most participants were female (N = 640; 85%), with an average age of 21 years (IQR 3). The analysis showed that students who were more determined to be vaccinated against COVID-19 were on average older, which was statistically significant at *p* < 0.001. Significant differences between genders was not established (Table 1).

Furthermore, a statistically significant difference (*p* < 0.001) was also found between students of different fields of study based on their willingness to be vaccinated. The findings indicate that a significant proportion of students across various fields of study remain uncertain regarding their vaccination preferences, with approximately one-third of students from each field reporting indecision. Among students who made a decision regarding their vaccination status, the results show that a slight majority of health science students (32.4%) express willingness to receive vaccination. In contrast, the majority of students from the natural sciences (36.7%) and social sciences (47.6%) report being unwilling to receive vaccination. Moreover, the data demonstrate that, among students who express willingness to receive vaccination, the majority belong to the health science field, whereas the majority of those unwilling to receive vaccination belong to the social science field (Table 1).

Statistically significant differences were also found between groups of students based on their willingness to be vaccinated and their different opinions about epidemiological measures (*p* < 0.001) and lockdown reintroduction (*p* = 0.002). The majority (51.7%) of students who thought that the implemented epidemiological measures were not effective were also not willing to be vaccinated. On the other hand, the majority of students (43.3%) who thought that the Civil Protection Directorate should reintroduce the lockdown were also willing to be vaccinated (Table 1).

For purposes of this analysis, information sources were grouped as being credible or less credible. Results show that a majority (54.5%) of students used less credible sources of information. Students who used credible sources of information had a more significant proportion of those willing to be vaccinated than those who used less credible sources or did not think about it. Furthermore, the majority of students who used less credible sources (40.7%) or did not think about it (48.4%) were unwilling to be vaccinated. These differences were statistically significant at *p* < 0.001 (Table 1).

On average, the most considerable adherence to epidemiological measures (median 9; IQR 2) and most tremendous confidence in the experts of the Civil Protection Directorate (median 7; IQR 3) were found among students who were willing to be vaccinated. Significant differences among other variables were not determined (Table 1).

A detailed analysis was conducted to identify differences between students studying health, natural, and social sciences. The results indicate that the majority of students did not contract COVID-19. However, among those who did contract the disease, the majority belonged to the health sciences field. Moreover, most students of natural and social sciences believed that the current epidemiological measures had not been effective in curbing the spread of the virus. Conversely, most students studying health sciences thought that these measures had been effective. Additionally, students of health sciences display greater adherence to epidemiological measures and have more trust in the experts at the Civil Protection Directorate of Croatia, as evidenced by statistically significant results at *p* < 0.001. While there were no significant differences observed between the sources of information used by students, the majority of those who utilized credible sources of information belonged to the health sciences field (Table 2).

A multiple binary logistic regression model was constructed to further establish the factors influencing the vaccination decision. The model was statistically significant, χ^2^ (25) = 94.519, *p* < 0.001, explained 17.1% of the variance (Nagelkerke R^2^ = 0.171), and correctly classified 74.7% of cases (Table 3).

Variables that were found to be predictive were gender, age, type of study field, opinion about the efficiency of previous epidemiological measures in the prevention of the spread of infection, opinion about reintroducing lockdown, and sources of information.

The model shows that each additional year of life contributes to a 6.9% increase in odds of being vaccinated. Furthermore, females had nearly 40% lower odds of being vaccinated than males (*p* = 0.035), and social science students had 47% lower odds of being vaccinated than health science students (*p* = 0.034). In addition, students who believed that previous epidemiological measures had not been effective in preventing the spread of infection had 52% lower odds of being vaccinated than students who had positive attitudes about the effectiveness of epidemiological measures (*p* = 0.001). Furthermore, compared to the same subgroup, students who did not know whether epidemiological measures had been effective had 43% lower odds of being vaccinated (*p* = 0.012). Students who believed that a lockdown was not necessary had 50% lower odds of being vaccinated than students who believed that reintroducing lockdown was necessary (*p* = 0.031). Finally, students who followed less credible sources of information had 36% lower odds of being vaccinated (*p* = 0.025), and those who did not think about it had 65% lower odds of being vaccinated (*p* = 0.001) compared to students who followed credible sources of information (Table 3).

## 4. Discussion

This study has shown that health education and health-related messages in the media are crucial in citizens’ lives, especially regarding vaccination against COVID-19. On almost all measurement variables related to the influence of various factors on willingness to vaccinate, health science students have more positive attitudes towards vaccination. They also have confidence in the relevant institutions, but they still use less credible scientific publications. Similar results have been shown in previous studies [26,27]. A systematic review that included 27 studies on rates of vaccination refusal against COVID-19 shows that students who attend college in non-health-related fields (non-health studies) have higher odds of refusing vaccination against COVID-19 compared to health science students [26].

The following factors have been found to strongly influence the decision to vaccinate: gender, age, type of study, respondents’ opinions about the effectiveness of interventions, the effectiveness of prohibitions, and the sources of information on which the decision to vaccinate is based. Previous studies have also shown that women have significantly lower vaccination odds [28,29,30,31]. An additional factor was age, which is also already established as a significant factor that influences vaccination decisions [32,33,34]. Furthermore, sources of information about vaccination [35], mistrust in vaccine safety often pointed out by media [36], and trust in government [37] have also been proved to be important factors in deciding on vaccination.

The majority (90.5%) of medical students at the Universities of Tanta and Kafrelsheikh, Egypt, saw the importance of the COVID-19 vaccine, 46% were hesitant to be vaccinated, and an equal percentage (6%) either accepted or refused the vaccine [38]. In addition, a study conducted in Croatia found that health school graduates can better critically evaluate health-related claims than their peers from high schools and other vocational schools. This shows that health education and the ability to evaluate health claims critically are significant factors in making sound health decisions [39], which is also supported by the findings of this study.

Previous studies have shown that vaccine hesitancy is mainly caused by the public’s perception of low safety, fear of new technologies and potential genetic mutations caused by vaccines, lack of effectiveness [40], and information insufficiency about the harmful effects of the vaccine [38].

Using reliable sources of COVID-19 information and building trust in vaccines through transparency and management of efficacy expectations are essential factors in this and previous studies [41]. Furthermore, the importance of transparent and plausible explanations regarding concerns about the speed and safety of vaccine development also proved to be a significant component in forming an opinion about vaccines [42]. It was also pointed out that, due to the various opinions presented in the media debates and discussions about the safety of the various vaccines, great attention should be paid to developing effective and consistent communication strategies [43]. However, while most studies have shown low vaccine hesitancy among health science students [42,44,45], some studies have shown no significant differences between health and non-health students in the factors contributing to vaccination hesitancy [17]. In addition, other factors influencing vaccination hesitancy and indecision change over time [46], in association with various factors such as strategies aimed at increasing awareness, restoring trust in healthcare professionals and health authorities, and limiting misinformation about vaccines [47]. This study has shown that health science students have more confidence in experts than other students, adhere more to government-recommended protective epidemiological measures, and believe that these measures effectively protect them from COVID-19.

Our study also had some limitations. First, this was a cross-sectional study, making it impossible to establish a causal relationship. Second, the data were collected using an online questionnaire because of the epidemic caused by the SARS-CoV-2 virus, which made it impossible to collect data traditionally. In addition, women predominate in our sample. As one of the possible reasons, men have a lower propensity to participate in online research than women [48]. According to the study of Park K., et. al., online survey participation rates tend to increase when the topic is attractive to the participants [49]. We believe that the low response rate in our survey (21.5%) was also influenced by the large number of requests for research related to COVID-19 that were carried out during that period. To address this issue, we sent reminders to encourage greater participation.

## 5. Conclusions

The skills of critical judgment of health information have proven to be one of the key factors in making all health decisions, including the decision about vaccination against COVID-19. However, the inability to identify reliable and relevant sources of information on vaccination, lack of adherence to the principles of evidence-based medicine, and prevailing distrust in governmental management of the pandemic all indicate the growing urgency to educate students about health and to raise their health literacy levels. Improving health literacy can be critical in health promotion and disease prevention. In addition, promoting health literacy among students raises social and health awareness among those future generations. Further studies can be conducted on the overall population to evaluate their willingness to receive potential new vaccines for emerging pandemics. By adopting this approach, we can effectively tackle upcoming public health challenges.

## Figures and Tables

**Table 1:** General characteristics of the sample (N = 752) according to preferences on question “Are you planning to be vaccinated against COVID-19?”.

Variables	Overall Sample N = 752	Are You Planning to Be Vaccinated against COVID-19?			*p*
		Yes	No	Do Not Know	
**Gender; N (%)**					
Male	112 (14.9)	41 (36.6)	36 (32.1)	35 (31.3)	0.058 †
Female	640 (85.1)	165 (25.8)	250 (39.1)	225 (35.2)	
**Age; median (IQR)**	21 (3)	22 (4)	21 (3)	21 (3)	**<0.001** #
**Field of study; N (%)**					
Health sciences	272 (36.2)	88 (32.4)	87 (32)	97 (35.7)	**<0.001 †**
Natural sciences	270 (35.9)	82 (30.4)	99 (36.7)	89 (33)	
Social sciences	210 (27.9)	36 (17.1)	100 (47.6)	74 (35.2)	
**University programs; N (%)**					
Undergraduate degree	636 (84.6)	172 (27.0)	246 (38.7)	218 (34.3)	0.689 †
Graduate degree	116 (15.4)	34 (29.3)	40 (34.5)	42 (36.2)	
**Place of residence; N (%)**					
Urban	515 (68.5)	139 (27)	188 (36.5)	188 (36.5)	0.563 **†**
Semi-urban	138 (18.4)	39 (28.3)	56 (40.6)	43 (31.2)	
Village	99 (13.2)	28 (28.3)	42 (42.4)	29 (29.3)	
**Living with N (%)**					
Alone	83 (11)	21 (25.3)	31 (37.3)	31 (37.3)	0.591 **†**
In a family with one more member/or partner	126 (16.8)	44 (34.9)	43 (34.1)	39 (31.0)	
In a family (more than two members)	456 (60.6)	119 (26.1)	179 (39.3)	158 (34.6)	
On the student campus	87 (11.6)	22 (25.3)	33 (37.9)	32 (36.8)	
**Attending classes; N (%)**					
In person (contact classes)	12 (1.6)	3 (25)	3 (25)	6 (50)	0.564 **††**
Online	136 (18.1)	37 (27.2)	55 (40.4)	44 (32.4)	
Combined (contact and online)	357 (47.5)	102 (28.6)	141 (39.5)	114 (31.9)	
Did not have classes (studying for exam deadlines)	247 (32.8)	64 (25.9)	87 (35.2)	96 (38.9)	
**Presence of chronic diseases; N (%)**					
Yes	92 (12.2)	29 (31.5)	32 (34.8)	31 (33.7)	0.617 †
No	660 (87.8)	177 (26.8)	254 (38.5)	229 (34.7)	
**COVID-19; N (%)**					
Yes	142 (18.9)	43 (30.3)	49 (34.5)	50 (35.2)	0.326 †
No	371 (49.3)	109 (29.4)	141 (38)	121 (32.6)	
Do not know	239 (31.8)	54 (22.6)	96 (40.2)	89 (37.2)	
**COVID-19 symptoms; N (%)**					
Asymptomatic disease	32 (4.3)	9 (28.1)	15 (46.9)	8 (25)	0.391 ††
Mild to moderate symptoms	131 (17.4)	35 (26.7)	49 (37.4)	47 (35.9)	
Severe symptoms	19 (2.5)	8 (42.1)	3 (15.8)	8 (42.1)	
I did not have COVID-19	570 (75.8)	154 (27)	219 (38.4)	197 (34.6)	
**Do you think that the epidemiological measures taken thus far have been effective in preventing the spread of the disease?; N (%)**					
Yes	269 (35.8)	101 (37.5)	63 (23.4)	105 (39)	**<0.001 †**
No	269 (35.8)	51 (19)	139 (51.7)	79 (29.4)	
Do not know	214 (28.5)	54 (25.2)	84 (39.3)	76 (35.5)	
**Do you think that the Civil Protection Directorate should reintroduce lockdown?; N (%)**					
Yes	60 (8)	26 (43.3)	18 (30)	16 (26.7)	**0.002 †**
No	628 (83.5)	154 (24.5)	252 (40.1)	222 (35.4)	
Do not know	64 (8.5)	26 (40.6)	16 (25)	22 (34.4)	
**Sources of information about COVID-19; N (%)**					
Credible sources of information *	220 (29.3)	79 (35.9)	60 (27.3)	81 (36.8)	**<0.001 †**
Less credible sources of information **	410 (54.5)	109 (26.6)	167 (40.7)	134 (32.7)	
I do not think about it	122 (16.2)	18 (14.8)	59 (48.4)	45 (36.9)	
**Adherence to the epidemiological measures; median (IQR)**	8 (2)	9 (2)	8 (4)	8 (7)	**<0.001** #
**Confidence in the experts of the Civil Protection Directorate of Croatia; median (IQR)**	5 (4)	7 (3)	3 (5)	6 (4)	**<0.001** #

†—χ^2^; ††—Fisher exact test; #—Kruskal–Wallis test; *—Credible sources of information (“Website of the Croatian Institute of Public Health (hzjz.hr)”, “Website of the Croatian Government (koronavirus.hr)”, “Scientific research articles”, “TV” (diary, information from the Civil Protection Directorate); **—Less credible sources of information (“Internet (in general)”, “Newspapers”, “Articles on the Internet (webpages: 24 h, indeks.hr, dnevnik.hr, Slobodna Dalmacija, Jutarnji list, or some other internet sources)”, “Via friends”).

**Table 2:** Differences between groups of students from different fields of study.

	Health Sciences (N = 272)	Natural Sciences (N = 270)	Social Sciences (N = 210)	*p*
**COVID-19; N (%)**				
Yes	63 (23.2)	48 (17.8)	31 (14.8)	**0.003 †**
No	144 (52.9)	119 (44.1)	108 (51.4)	
Do not know	65 (23.9)	103 (38.1)	71 (33.8)	
**University programs; N (%)**				
Undergraduate degree	272 (100)	204 (75.6)	160 (76.2)	**<0.001 ††**
Graduate degree	0 (0)	66 (24.4)	50 (23.8)	
**Attending classes; N (%)**				
In person (contact classes)	0 (0)	12 (4.4)	0 (0)	**<0.001 ††**
Online	89 (32.7)	29 (10.7)	18 (8.6)	
Combined (contact and online)	183 (67.3)	93 (34.4)	81 (38.6)	
Did not have classes (studying for exam deadlines)	0 (0)	136 (50.4)	111 (52.9)	
**Are you planning to be vaccinated against COVID-19?**				
Yes	88 (32.4)	82 (30.4)	36 (17.1)	**0.001 †**
No	87 (32)	99 (36.7)	100 (47.6)	
Do not know	97 (35.7)	89 (33)	74 (35.2)	
**Do you think that the epidemiological measures taken thus far have been effective in preventing the spread of the disease?; N (%)**				
Yes	117 (43)	82 (30.4)	70 (33.3)	**0.004 †**
No	76 (27.9)	106 (39.3)	87 (41.4)	
Do not know	79 (29)	82 (30.4)	53 (25.2)	
**Sources of information about COVID-19; N (%)**				
Credible sources of information *	92 (33.8)	67 (24.8)	61 (29)	0.207 †
Less credible sources of information **	138 (50.7)	154 (57)	118 (56.2)	
I do not think about it	42 (15.4)	49 (18.1)	31 (14.8)	
**Adherence to the epidemiological measures; median (IQR)**	9 (3)	8 (3)	8 (2)	**<0.001 #**
**Confidence in the experts of the Civil Protection Directorate of Croatia; median (IQR)**	6 (4)	5 (5)	5 (4)	**<0.001 #**

†—χ^2^; ††—Fisher exact test; #—Kruskal–Wallis test; *—Credible sources of information (“Website of the Croatian Institute of Public Health (hzjz.hr)”, “Website of the Croatian Government (koronavirus.hr)”, “Scientific research articles”, “TV” (diary, information from the Civil Protection Directorate); **—Less credible sources of information (“Internet (in general)”, “Newspapers”, “Articles on the Internet (webpages: 24 h, indeks.hr, dnevnik.hr, Slobodna Dalmacija, Jutarnji list, or some other internet sources)”, “Via friends”).

**Table 3:** The results of multiple binary logistic regression used to predict students’ willingness to be vaccinated.

Variables	Odds Ratio (95% CI)	*p*
**Age** (years)	1.069 (1.033–1.105)	**<0.001**
**Women** (men are referent group)	0.594 (0.374–0.944)	**0.027**
**Field of study** (Health sciences is referent group)		
Natural sciences	1.175 (0.697–1.981)	0.545
Social sciences	0.560 (0.313–1.000)	**0.050**
**Graduate degree** (undergraduate degree is referent group)	1.096 (0.651–1.846)	0.729
**Place of residence** (urban is referent group)		
semi-urban	1.082 (0.683–1.715)	0.736
village	1.160 (0.682–1.975)	0.584
**Living with** (alone is referent group)		
In a family with one more member/or partner	1.272 (0.652–2.479)	0.481
In a family (more than two members)	0.966 (0.536–1.742)	0.908
On the student campus	1.192 (0.562–2.531)	0.647
**Attending of classes** (In person (contact classes) are referent group)		
Online	0.644 (0.143–2.905)	0.567
Combined (contact and online)	0.953 (0.223–4.069)	0.948
Did not have classes (studying for exam deadlines)	1.093 (0.259–4.607)	0.903
**Presence of chronic diseases** (“yes” is referent group)	0.911 (0.542–1.532)	0.726
**COVID-19** (“yes” is referent group)		
No	1.236 (0.459–3.327)	0.676
Do not know	0.917 (0.372–2.265)	0.852
**Severity of COVID-19 symptoms** (Asymptomatic are referent group)		
Mild to moderate symptoms	0.769 (0.290–2.040)	0.597
Severe symptoms	1.727 (0.431–6.927)	0.441
I did not have COVID-19	0.738 (0.263–2.070)	0.563
**Effectiveness of epidemiological measures taken thus far** (“yes” is referent group)		
No	0.477 (0.311–0.732)	**<0.001**
I do not know	0.590 (0.385–0.904)	**0.015**
**Need for reintroduce lockdown** (“yes” is referent group)		
No	0.501 (0.272–0.921)	**0.026**
Do not know	0.837 (0.374–1.877)	0.667
**Sources of information** (Credible sources are referent group)		
Less credible sources of information	0.640 (0.435–0.941)	**0.023**
I do not think about it	0.367 (0.198–0.679)	**<0.001**

## Data Availability

The data presented in this study are available on request from the corresponding author.
